# Acupuncture as adjunctive treatment for linezolid-induced peripheral neuropathy: a case series report

**DOI:** 10.3389/fneur.2024.1388544

**Published:** 2024-06-21

**Authors:** Yuping Mo, Fan Nie, Jiahui Wu, Linna Li, Zhu Zhu, Guofang Deng, Liang Fu

**Affiliations:** ^1^Traditional Chinese Medicine Department, Shenzhen Third People's Hospital, Southern University of Science and Technology, National Clinical Research Center for Infectious Disease (Shenzhen), Guangdong Provincial Clinical Research Center for Infectious Diseases (Tuberculosis), Shenzhen Clinical Research Center for Tuberculosis, Shenzhen, China; ^2^National Clinical Research Center for Infectious Disease (Shenzhen), Shenzhen, China; ^3^Guangzhou University of Chinese Medicine, Guangzhou, China; ^4^Pulmonary Diseases Department, Shenzhen Third People's Hospital, Southern University of Science and Technology, National Clinical Research Center for Infectious Disease (Shenzhen), Guangdong Provincial Clinical Research Center for Infectious Diseases (Tuberculosis), Shenzhen Clinical Research Center for Tuberculosis, Shenzhen, China

**Keywords:** peripheral neuropathy, acupuncture, linezolid, case report, multidrug-resistant tuberculosis

## Abstract

**Background:**

The treatment of multidrug-resistant tuberculosis (MDR-TB) and pre-extensively drug-resistant tuberculosis (pre-XDR-TB) remains challenging due to the limited availability of effective drugs. Linezolid has emerged as a promising therapeutic option for these cases. However, its long-term use can lead to complications such as peripheral and optic neuropathies. Acupuncture, a cornerstone of traditional Chinese medicine, has been shown to be effective in the treatment of peripheral neuropathy (PN). This study examines the potential benefits of acupuncture in the treatment of linezolid-induced peripheral neuropathy (LIPN).

**Methods:**

Four patients, aged 27 to 60 years, diagnosed with LIPN, underwent daily acupuncture treatments. The main endpoint was to assess the efficacy of acupuncture in reducing neuropathic pain associated with LIPN in patients. This was primarily measured using changes in the Short Form McGill Pain Questionnaire (SF-MPQ) scores before and after acupuncture treatment.

**Results:**

Three of the patients experienced significant symptom remission, while one experienced marginal improvement. Treatments ranged from 7 to 18 sessions. Specifically, the first patient reported substantial relief with a score reduction from 33 to 13; the second patient observed minimal change; the third patient’s score decreased dramatically from 10 to 2 after eight sessions; the last patient had a score reduction from 21 to 12 after five sessions, but did not continue treatment for a second assessment.

**Conclusion:**

Acupuncture is a promising therapeutic approach for LIPN. However, larger and more thorough studies are needed to determine its full potential.

## Introduction

Tuberculosis (TB), caused by infection with *Mycobacterium tuberculosis* (MTB), is an ancient disease that remains a leading infectious cause of death worldwide (1, 2). It poses a significant threat to global public health security. Pulmonary tuberculosis (PTB), which mainly affects adults and children, serves as a major source of transmission of the infection. Therefore, accurate diagnosis and timely, effective treatment are critical to any successful TB control strategy (3).

In 2021, tuberculosis will claim approximately 1.6 million lives, according to the World Health Organization’s (WHO) 2022 global tuberculosis report (4). A major driver of TB-associated mortality is antimicrobial resistance (AMR), with MTB recognized as a priority pathogen in AMR surveillance (5). Multidrug-resistant tuberculosis (MDR-TB) manifests as resistance to two essential first-line drugs: rifampicin and isoniazid. Pre-extensively drug-resistant tuberculosis (pre-XDR-TB) is a more severe form of MDR-TB with extra resistance to fluoroquinolones (FQ). In contrast to the high cure rate of drug-sensitive TB, drug-resistant TB has lower cure rates and increased mortality (1).

Linezolid (Lzd) is a synthetic zolidinone antibacterial developed primarily to combat infections caused by vancomycin-resistant gram-positive cocci. After receiving Food and Drug Administration (FDA) approval in 2000, China introduced linezolid in 2007, recognizing its potent antimycobacterial properties. By 2018, the WHO had classified linezolid as a Group A drug for the treatment of MDR-TB. Given that MDR-TB treatment often lasts 18 to 24 months, concerns about its long-term side effects have grown. In particular, linezolid-induced peripheral neuropathy (LIPN) can severely affect patients’ quality of life and, in severe cases, require discontinuation of the drug, leading to TB treatment failure.

Peripheral neuropathy (PN) is characterized by damage to the peripheral nervous system due to an inherent lesion or dysfunction. Polyneuropathy involves multiple nerves, while mononeuropathy is limited to a single nerve. Often associated with painful paresthesias, PN remains a significant therapeutic challenge. Acupuncture, an ancient treatment from Traditional Chinese Medicine (TCM), is experiencing a surge in global adoption. Numerous clinical studies have demonstrated its efficacy in alleviating drug-induced peripheral neuropathy or neurotoxic symptoms such as pain, numbness and paresthesia, thereby improving patients’ quality of life while ensuring safety.

Having previously reported a related case study, our research group was encouraged by the tangible benefits of acupuncture. This article aims to highlight the efficacy of acupuncture in improving neuropathic pain in clinical practice.

## Methods

### Patient selection and pain assessment tool

Patients diagnosed with LIPN and undergoing lower extremity anesthesia were enrolled in our study (Table 1). Prior to beginning acupuncture treatment, each patient was fully informed of the procedure and its safety after treatment. With full understanding, they consented to receive daily acupuncture sessions at the acupuncture department.

**Table 1:** Baseline demographic, clinical, and biological characteristics of four MDR-TB patients.

Characteristics	Case 1	Case 2	Case 3	Case 4
Age (year)	27	60	38	29
Sex	Men	Women	Women	Men
Therapeutic medications				
Lzd (Linezolid)	Yes	Yes	Yes	Yes
Bdq (Bedaquinoline)	Yes	Yes	Yes	Yes
Cs (Cycloserine)	Yes	Yes	Yes	No
Cfz (Clofazimine)	Yes	No	Yes	No
Z (Pyrazinamide)	No	No	Yes	Yes
Mfx (Moxifloxacin)	No	Yes	No	No
Lvfx (Levofloxacin)	No	No	No	Yes
Pth (Protionamide)	Yes	No	No	No
Comorbidities at Anti-TB initiation				
Diabetes	No	Yes	No	No

Pain intensity was assessed both before and after acupuncture treatment using the short form of the McGill Pain Questionnaire (SF-MPQ) (6). This questionnaire consists of three different sections: Pain Rating Index (PRI): This assesses both the sensory and emotional aspects of pain. Scores range from 0, indicating no pain, to 3, indicating severe pain. Visual Analog Scale (VAS): Part of the SF-MPQ used to measure overall pain perception. Present Pain Intensity (PPI): Another component of the SF-MPQ used to assess immediate pain intensity. The combined scores from these tools served as a measure of pain severity, with higher scores indicating more intense pain.

### Treatment protocol

A licensed acupuncturist administered the treatment. The patient was positioned supine for optimal comfort. The acupuncture treatment protocol for LIPN was based on the classical meridian system associated with the condition. Three main meridians were targeted: Stomach meridian of foot-yangming, Spleen channel of foot-taiyin, Kidney meridian of foot-shaoyin. Based on professional expertise, the following acupoints on both legs were selected: Zusanli (ST 36), Sanyinjiao (SP 6), Taixi (KI 3), Yongquan (KI 1), Bafeng (EX-LE10).

**Figure 1:**
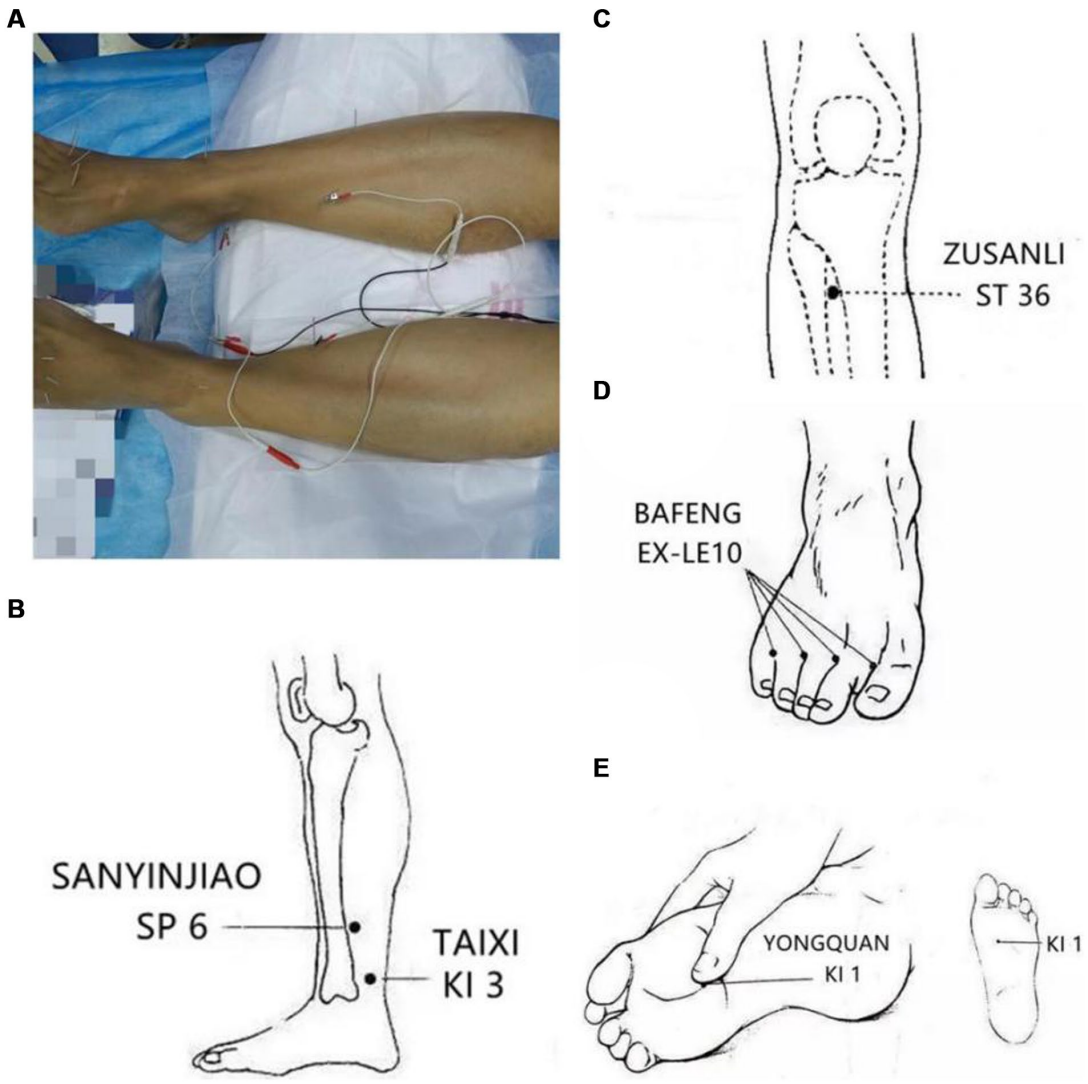
The acupoints of the treatment: **(A)** The patient on acupuncture, SP6 and KI 3 were connected to electroacupuncture equipment for 30 min. **(B)** The main acupoints for the patient, bilateral Sanyinjiao (SP 6) and Taixi (KI 3). **(C)** The main acupoints for the patient, bilateral Zusanli (ST 36). **(D)** The main acupoints for the patient, bilateral Bafeng (EX-LE10). **(E)** The main acupoints for the patient, bilateral Yonquan (KI 1).

The practitioner used sterile acupuncture needles, specifically 0.30 × 25 mm and 0.18 × 13 mm (Huacheng, Beijing Keyuan Medical Device Manufactory Co., Ltd., Beijing, China). The penetration depth for each point was as follows: ST 36: 1–2 cun, SP 6: 1–1.5 cun, KI 3: 0.5–0.8 cun, KI 1: 0.5 cun, EX-LE10: 0.5–0.8 cun. After reaching the desired depth, a sensation known as “deqi” was sought. The needles positioned at SP 6 and KI 3 were then connected to an electroacupuncture device for a 30-min session (Figure 1). The device was set to emit a continuous wave, and the stimulation intensity was adjusted according to the patient’s comfort level.

## Results

**Figure 2:**
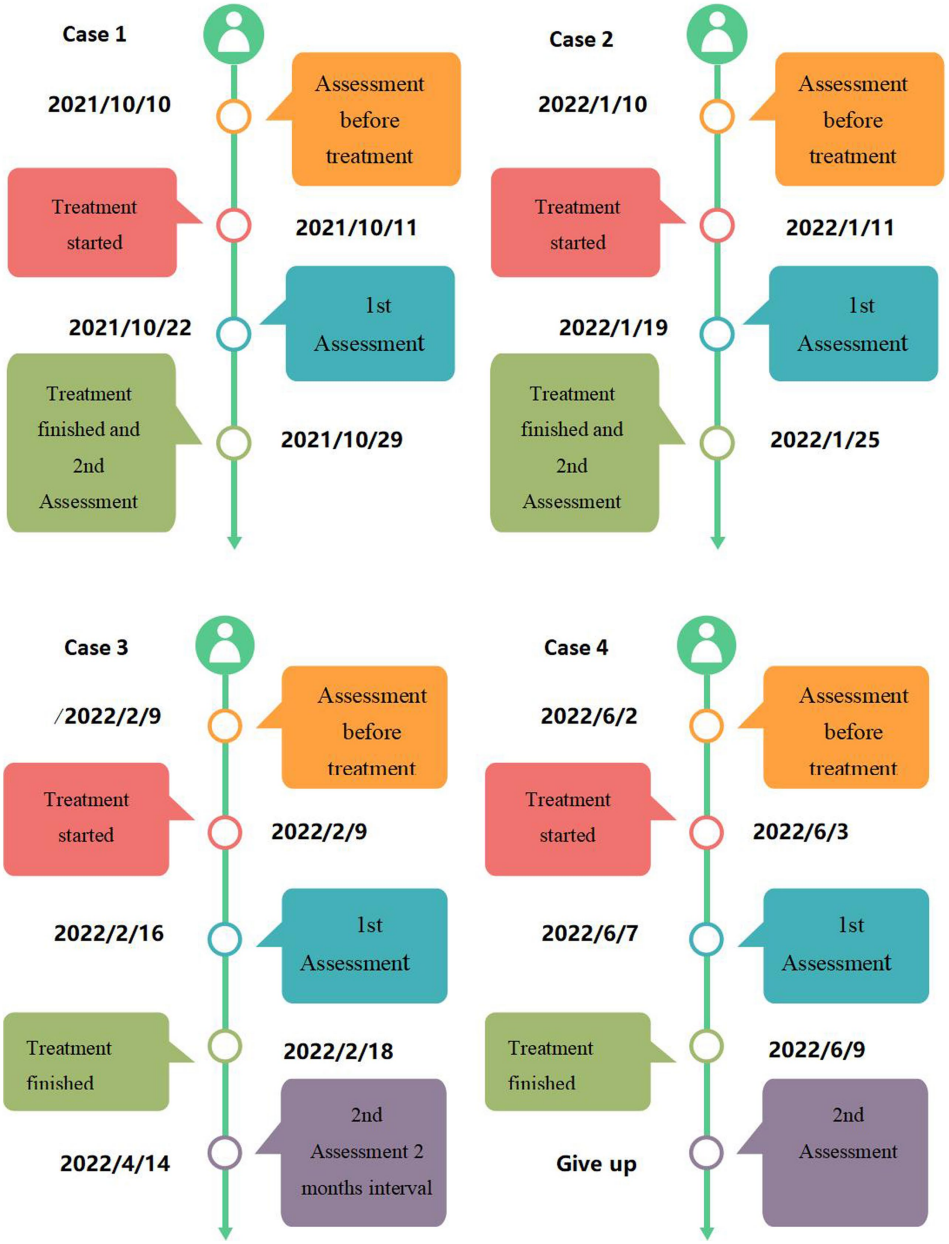
The timeline of the acupuncture treatments and assessments of the four patients.

**Figure 3:**
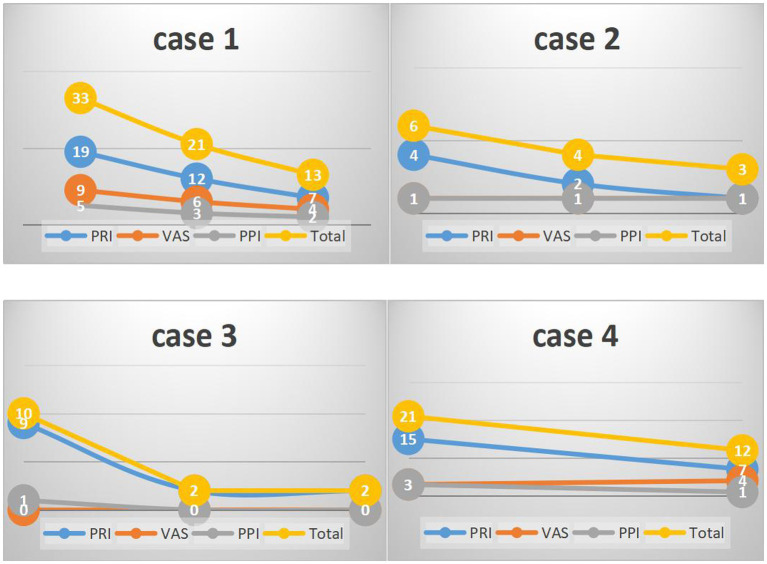
The result of the four patients’ SF-MPQ scores. PRI, pain rating index; VAS, visual analogue scale; PPI, present pain intensity.

A cohort of four patients, two males and two females, between the ages of 27 and 60 years were followed in this study. The duration of treatment varied between patients and ranged from 7 to 18 sessions (Table 2). Their treatment durations (Figure 2) and results (Figure 3) were as follows:

**Table 2:** The results of four patients’ SF-MPQ scores.

Patient No.	Gender	Age	Complication	Clinical courses	Initial SF-MPQ score	1st assessment score	2nd assessment score
1	Male	27	No	18 sessions	33	21 (11 sessions)	13 (18 sessions)
2	Female	60	Diabetes	15 sessions	6	4 (9 sessions)	3 (15 sessions)
3	Female	38	No	10 sessions	10	2 (8 sessions)	2 2 months interval
4	Male	29	No	7 sessions	21	12 (5 sessions)	Give up

Patient One, a male (specific age not given), began treatment with an initial SF-MPQ score of 33. After 11 therapy sessions, his score improved to 21. By the 18th session, he reported significant comfort and was able to walk for extended periods without discomfort. As a result, his SF-MPQ score was further reduced to 13, indicating significant symptom relief.

Patient Two, a 60-year-old female with a history of diabetes, began her treatment with an SF-MPQ score of 6. After 9 therapy sessions, her score dropped to 4. By the 15th session, there was a slight further improvement, resulting in a score of 3. Although there was a reduction, the relief was relatively modest compared to the others.

Patient Three, a 38-year-old woman, seemed to respond particularly well to acupuncture treatment. She started with an SF-MPQ score of 10, which dramatically decreased to 2 after only 8 sessions. Notably, this score remained at 2 two months later without further treatment, demonstrating the sustained effectiveness of the therapy for her.

Patient Four, a 29-year-old male, participated in 7 therapy sessions. He began treatment with an SF-MPQ score of 21. After five sessions, his score improved significantly to 12. However, it should be noted that he did not participate in a second assessment.

It is evidently to finger out the acupuncture therapy of 4 patients take place within the period of drug therapy according to Table 3 and Figure 2. This sentence implies one meaning: acupuncture effects in 4 patients are real, not placebo effects. If acupuncture treatment takes place when the drug therapy finished or one/two weeks later. It is difficult to distinguish whether the patients are self-cured or recovered from acupuncture therapy.

**Table 3:** The timeline of the drug therapy for the cases.

	The time point to begin drug therapy	The time point to start acupuncture	The time point to end acupuncture	The time point to end drug therapy
Case 1	April 8th, 2021	Oct 11th, 2021	Oct 29th, 2021	April 19th, 2023
Case 2	Sep 11th, 2021	Jan 11th, 2022	Jan 25th, 2022	Mar 16th, 2022
Case 3	Sep 11th, 2021	Feb 9th, 2022	Feb 18th, 2022	July 11th, 2022
Case 4	Feb 9th, 2022	Jul,3rd, 2022	Jul 9th, 2022	Nov 10th, 2022

## Discussion

This collection of case studies looks into how effective and safe acupuncture might be as a treatment for PN. PN mostly affects the lower legs and feet and can cause a range of symptoms, including pain, numbness, tingling, and burning. Many conditions can lead to PN, including diabetes, thyroid issues, vitamin B12 deficiency, excessive alcohol use, chemotherapy, and HIV. Traditionally, full recovery from PN is seen as challenging, if not impossible. However, some treatments, as shown in our study, offer hope for those with LIPN (7–9). Several studies from around the world have pointed out potential neurological risks with the drug linezolid (10). This underlines the importance of early treatment for LIPN.

Acupuncture, a traditional Chinese treatment, is based on the idea that our health depends on a steady flow of energy, or “qi.” If this flow is blocked, it can lead to illness. So, rather than focusing only on the disease itself, acupuncturists look to restore this flow. While not many studies have focused on how well acupuncture works for LIPN, our results, combined with what we have seen in practice, suggest it might be a useful approach.

Acupuncture, integral to TCM, effectively manages LIPN by promoting and tonifying qi and blood. This approach is particularly beneficial for alleviating LIPN symptoms by directing healing energies to affected areas, crucial for patients with chronic conditions like MDR-TB. In TCM, acupuncture is seen as a means to regulate “qi,” or body balance. Advances like electro-acupuncture (EA) enhance these effects by influencing neurological pathways and inflammatory processes. Acupuncture affects both peripheral and spinal cord levels, involving neurotransmitters such as Caspase-1, TNF-α, IL-1β, and IL-6 (11–14), which balance pro-inflammatory and anti-inflammatory responses. Additionally, receptors like CB2R, TLR4, TRPV1, and opioid receptors are involved in various signaling pathways (11–14), highlighting the complex mechanisms of acupuncture. Electroacupuncture specifically helps reduce neurological hyperalgesia and inflammatory cytokines (15), indicating that different acupuncture techniques engage distinct biological processes. This complexity suggests that further research is needed to fully understand how both traditional and modern acupuncture methods contribute to therapeutic outcomes.

The timing of SF-MPQ evaluations in our study was adapted to individual patient responses and logistical constraints, reflecting the personalized approach of our acupuncture treatment. Evaluations were scheduled to coincide with peak therapeutic effects as observed by clinicians, allowing for the most significant symptom changes to be documented. This flexibility accommodated patients’ varying abilities to attend sessions, ensuring better adherence and more accurate assessment of pain relief. Clinical decisions on the timing of evaluations were based on observed improvements, ensuring that each assessment was relevant and timely. While this approach introduced some variability, it was essential for tailoring the treatment to individual needs, a key aspect of effective clinical practice.

However, our study does have some other limitations. We mainly used the SF-MPQ to measure pain, which might not give a full picture. It could be helpful to also use other tools like electromyograms, which measure muscle activity, and feedback from patients. Also, our study only included a small number of cases, so we should be careful about drawing broad conclusions. Other factors, like how well the patient and doctor communicate, or the patient’s overall health, can also influence results. Despite the marked efficacy observed in the four cases, individual responses to treatment varied. While acupuncture undeniably enhances body metabolism, the extent of LIPN improvement may be influenced by individual metabolic capacity and other unknown factors. Since we did not have a control group (for example, patients treated with a placebo instead of acupuncture), it’s hard to say for sure that the improvements we saw were all because of the acupuncture.

## Conclusion

The presented case studies delved into the potential benefits of acupuncture for LIPN patients. While individual outcomes varied, the majority highlighted the therapeutic advantages of acupuncture in managing LIPN. Given these encouraging results, promoting broader use of acupuncture can potentially help a larger segment of LIPN sufferers. Acupuncture, with its deep historical roots, still holds significant value in today’s medical practices. In the dynamic world of medicine, the confluence of traditional wisdom and modern innovation often provides the most holistic solutions. Embracing both may indeed pave the way for profound medical advancements.

## Data availability statement

The datasets presented in this article are not readily available because of ethical and privacy restrictions. Requests to access the datasets should be directed to the corresponding authors.

## Ethics statement

The studies involving humans were approved by the Ethics Committee of Shenzhen Third People's Hospital. The studies were conducted in accordance with the local legislation and institutional requirements. The participants provided their written informed consent to participate in this study. Written informed consent was obtained from the individual(s) for the publication of any potentially identifiable images or data included in this article.

## Author contributions

YM: Writing – original draft, Writing – review & editing. FN: Writing – original draft. JW: Data curation, Writing – review & editing. LL: Project administration, Writing – review & editing. ZZ: Project administration, Writing – review & editing. GD: Funding acquisition, Investigation, Resources, Supervision, Writing – review & editing. LF: Conceptualization, Writing – original draft, Writing – review & editing.
